# MSGP: the first database of the protein components of the mammalian stress granules

**DOI:** 10.1093/database/baz031

**Published:** 2019-03-01

**Authors:** Catarina Nunes, Isa Mestre, Adriana Marcelo, Rebekah Koppenol, Carlos A Matos, Clévio Nóbrega

**Affiliations:** 1 Department of Biomedical Sciences and Medicine, University of Algarve, Faro, Portugal; 2 Centre for Biomedical Research, University of Algarve, Faro, Portugal; 3 Center for Neuroscience and Cell Biology, University of Coimbra, Coimbra, Portugal; 4 Algarve Biomedical Center, University of Algarve, Faro, Portugal

## Abstract

In response to different stress stimuli, cells transiently form stress granules (SGs) in order to protect themselves and re-establish homeostasis. Besides these important cellular functions, SGs are now being implicated in different human diseases, such as neurodegenerative disorders and cancer. SGs are ribonucleoprotein granules, constituted by a variety of different types of proteins, RNAs, factors involved in translation and signaling molecules, being capable of regulating mRNA translation to facilitate stress response. However, until now a complete list of the SG components has not been available. Therefore, we aimer at identifying and linting in an open access database all the proteins described so far as components of SGs. The identification was made through an exhaustive search of studies listed in PubMed and double checked. Moreover, for each identified protein several details were also gathered from public databases, such as the molecular function, the cell types in which they were detected, the type of stress stimuli used to induce SG formation and the reference of the study describing the recruitment of the component to SGs. Expression levels in the context of different neurodegenerative diseases were also obtained and are also described in the database. The Mammalian Stress Granules Proteome is available at https://msgp.pt/, being a new and unique open access online database, the first to list all the protein components of the SGs identified so far. The database constitutes an important and valuable tool for researchers in this research area of growing interest.

## Introduction

Cells are exposed to different stress stimuli that they need to overcome ensuring cell survival. To manage stress, cells have several mechanisms ranging from repair pathways to apoptosis triggering, if cells fail to overcome the stress. Growing evidence suggests that a persistent cellular stress state might underlie an enhanced susceptibility to aging or aging-related diseases, like neurodegenerative disorders or cancer (1).

The assembly of stress granules (SGs) represents a conservative component of the cellular response to stress. SGs are ribonucleoprotein granules that appear when eukaryotic cells are exposed to certain types of stimuli such as endoplasmic reticulum stress, heat shock, hypoxia, arsenite, viral infection or overexpression of specific RNA-binding proteins (RBPs) (2). SGs are transiently formed upon cellular stress, and their disassembly occurs when the cellular stressor is removed. The canonical SG assembly pathway is triggered by the phosphorylation of eIF2*α* leading to the inhibition of translation, and thereby creating a pool of mRNAs stalled in translation initiation, translation initiation factors, RBPs and ribosomal units (3). SG assembly is key to cell survival as these foci inhibit apoptosis through reduction of reactive oxygen species (ROS), sequestration of signaling molecules and stabilization of mRNAs of anti-apoptotic factors (4). Under stress conditions, global translation is reduced, and SGs are thought to function in the triage of repressed mRNAs, allowing a focused translation of proteins critical to overcome stress (5). Additionally, stalled mRNAs in SGs are protected from degradation during stress and can rapidly re-enter the translational pool once stress is overcome and they are released (6). Despite these important functions in translation and several others described, the complete functions of SGs are not yet understood.

The molecular composition of SG core is based in stalled mRNA transcripts, poly(A) mRNAs, RBPs, translation initiation factors, proteins with predicted low-complexity domains and small (40S) ribosomal units (7). Due to their frequent presence in SGs, some proteins, are commonly used as SG markers, including several eukaryotic initiation factors, poly(A)-binding protein 1 (PABP1), T-cell intracellular antigen 1 (TIA-1), TIA-1-related protein (TIAR), Ras GTPase-activating protein-binding protein 1 (G3BP1) and ataxin-2 (8). Nevertheless, SG composition changes during the stress response and is also different according to the type of stress or cell (9). In fact, recently, it was found that ~20% of SG components diversity is dependent on the stress and the cell type (10).

Growing and recent evidence implicates SGs in the context of human disease, namely in cancer (2) and in neurodegenerative disorders (11). For example, in cancer, SGs were found in different tumors with different histological origins (12–14). In the same line, in Alzheimer’s disease (AD) several SG components accumulate in affected cells and colocalize with pathogenic tau (15, 16). We also showed that, in the context of another neurodegenerative disease - Machado–Joseph, the SG component ataxin-2 is downregulated, contributing decisively to the pathology, whereas its overexpression ameliorates the disease phenotype (17). On the other hand, antisense oligonucleotides-mediated ATXN2 silencing was successful in reducing neuropathological abnormalities in spinocerebellar ataxia type 2 and amyotrophic lateral sclerosis animal models (18, 19). Additionally, SGs could also be implicated in the normal aging process, as a reduction in the expression of several SG components with age, especially RBPs, has been described (20).

A database consists in a storage of information that can be easily accessed and that is regularly managed and updated. Therefore, databases serve as an important tool for research and, accordingly, the number of databases has been increasing in the past years (21). Despite the growing interest in SG research and several reviews on the topic, there is still a lack of resources for their study. There are several important databases on RBPs, focusing on different aspects of their structure or function, although they do not address the RBPs’ role/presence in SGs (22, 23). SGs were originally described in tomato cell lines submitted to heat shock (24), and since then several studies demonstrated the recruitment of different proteins to SGs. However, the complete list of SG components is unknown. Thus, we generated electronic resources in the form of Excel-based databases/tables containing all the protein components recruited to SGs that have been described so far. These data were the basis for the development of an online database available at https://msgp.pt/, which we now present. The database curates general information about all the protein components of SGs described so far in mammalian cells. The platform provides a new and unique resource for the SG research field, collecting and storing for the first time and in the same place all the information on the SG protein components.

## Material and methods

### Components identification and curation

We curated the published literature available in different databases (like for example PubMed) covering all the SG protein components described in studies using mammalian cells. Several keyword combinations were used, such as stress granules AND mammals or stress granules AND sodium arsenite. Each study describing the recruitment of different proteins to SGs was double checked, and the type of cell, stimulus used and effective recruitment of the component to the SGs were annotated and confirmed. Additionally, for all the identified and validated SG components several details were gathered from public databases, including the protein abbreviation, gene ID, chromosomal location, Uniprot ID, molecular function, subcellular localization, original study describing its recruitment to SGs (along with the cell type and stimulus used), RBP classification [according to (25)], identity as autophagy-related proteins [according to (26)] and the OMIM details for their possible implication in human genetic disorders. All these details were gathered in the form of Excel-based databases/tables.

### Gene expression data analysis

The GEO Expression Omnibus public database was used to find studies describing gene expression data in different neurodegenerative diseases. From the found studies, three were chosen based on the high number of sampled individuals, as well as on the type of neurodegenerative disease studied.

Expression profiles for all the identified and curated SG components were extracted from a transcriptome data set of human brain biopsy tissue sample, covering subjects with AD, Huntington’s disease (HD) and healthy controls [GSE33000; (27)]; subjects with Parkinson’s disease (PD) and healthy controls (GSE28894); and subjects with amyotrophic lateral sclerosis (ALS) and healthy controls [GSE4595; (28)]. We analyzed the original expression data using the Geo2R web tool (http://www.ncbi.nlm.nih.gov/geo/geo2r/) comparing the different groups: AD versus controls, HD versus controls, PD versus controls and ALS versus controls. An adjusted *P* < 0.05 accessed the SG components whose expression was statistically different between groups. Adjustments were made to correct the occurrence of false positive results, using the Benjamini and Hochberg false discovery rate method.

### Software tools and database implementation

The Mammalian Stress Granules Proteome (MSGP) is an online and open access database. The website was implemented using the Wordpress system, and the database was build using ‘custom fields’, based on the open source tool ‘Elementor Page Builder’. The database includes, also, other important features like custom listing profile for each protein, custom fields with editing capability for each protein, highly customized GeneID cards, protein listing quick view, breadcrumbs navigation, custom dashboard for front and end users and customized and multiple IDs for each protein. We also included >50 widgets ready to use on the database (keeping in mind its future expansion), integrated in a clean system, compatible with PHP version 5.5+, and we used minified and combined assets to reduce the amount of http requests and enhance load time and site performance. The system was built using Vue.js JavaScript framework and we programmed clean and well-structured code, to facilitate access to the data. The MSGP platform was also conceived to be responsive, working on all types of devices (mobile phones, tablets, computers etc.) and integrating the future functionality of user sign in/registration.

## Results

### Database structure

**Figure 1:**
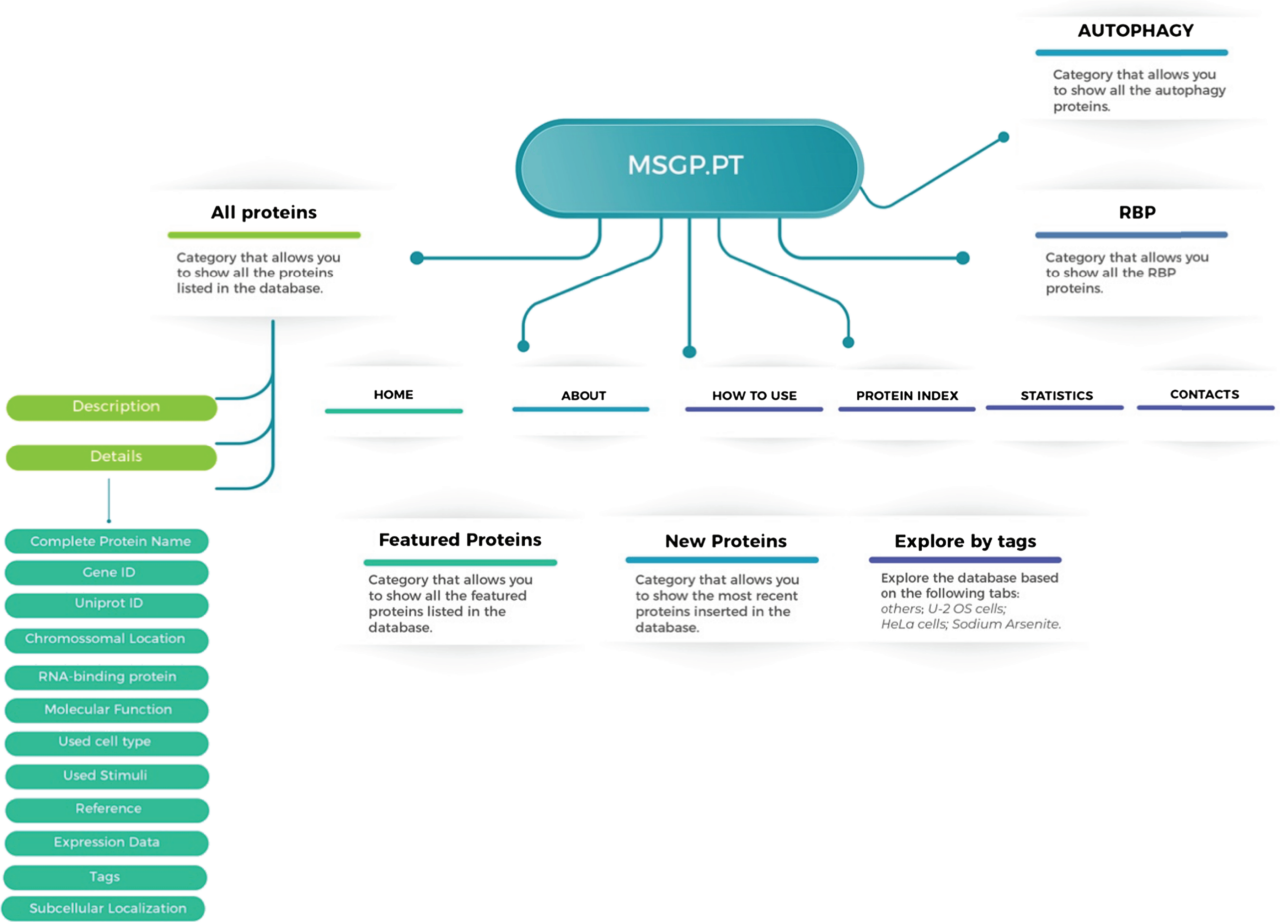
Structure of the MSGP database, depicting the main sections, different filters, tags and forms to explore and retrieve the information stored in the database.

The MSGP database was developed in a highly customized way, allowing the introduction of several important features and future uses. The structure and navigation were planned to work in a very intuitive and user-friendly manner. Moreover, a ‘How to use’ section where database users can find a tutorial showing the search process as well as the system navigation, especially in each SG component-specific page, was also created. The MSGP database contains primarily the 464 proteins identified as components of the SGs, which can be retrieved through different pages from the database website or from a general search. The structure of the database is described in Figure 1, providing three major sections: All proteins, grouping the information and details on the 464 proteins identified; the RBP section, where all SG components classified as RBPs are grouped; and the Autophagy section, where all SG components that belong to the autophagy pathway are grouped. Additionally, the database has a page ‘Protein Index’ where all the SG components are listed alphabetically. The database allows further exploring of the identified and curated components, in the `Featured Proteins' section or in the `New Proteins' section, where the proteins most recently included in the database are added. Furthermore, the database allows exploring the listed proteins according to different tags, such as sodium arsenite, U-2 OS or Hela cells.

### Database content

**Figure 2:**
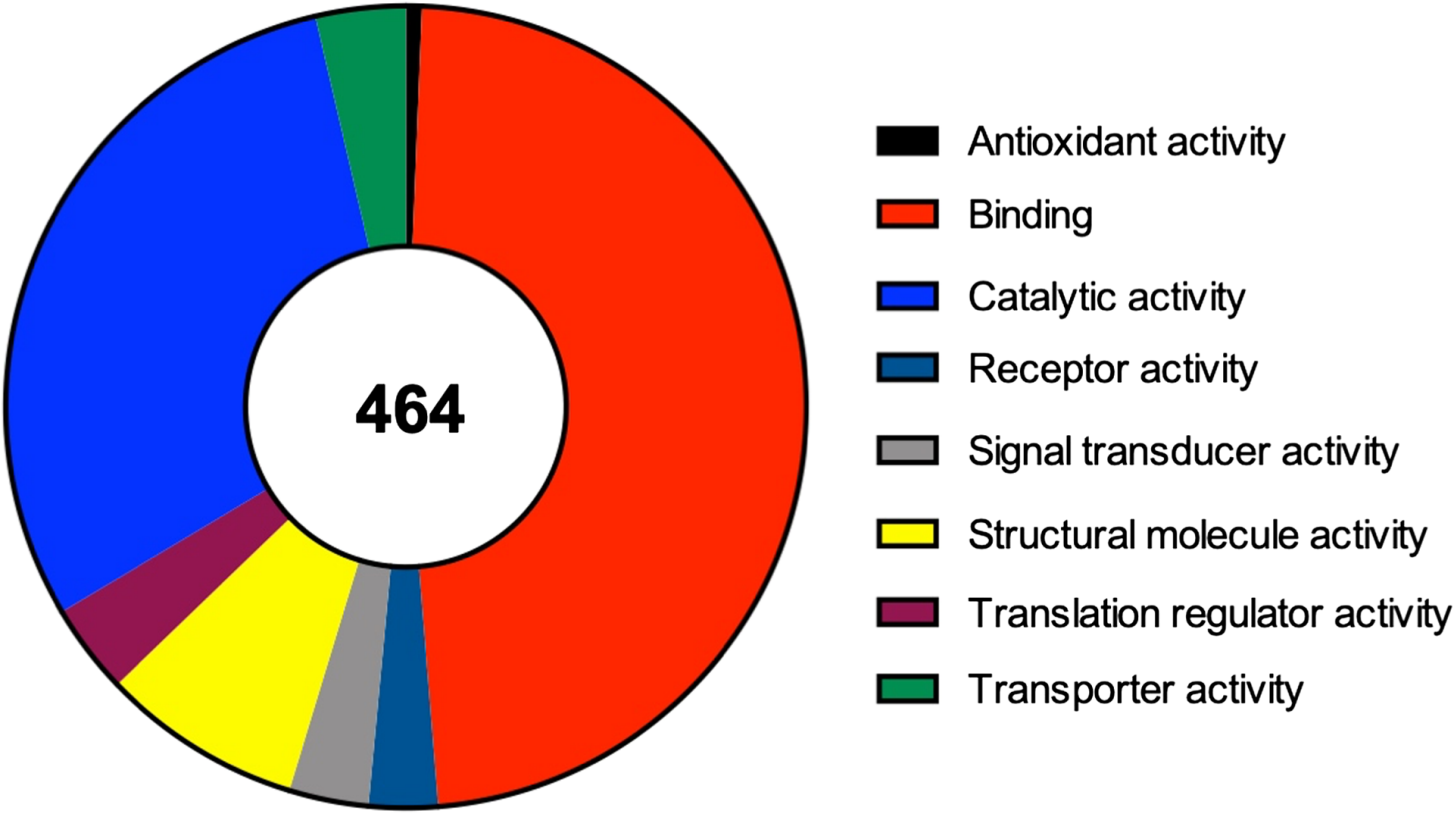
Molecular function of the 464 proteins currently identified as components of mammalian SGs, according to a gene list analysis by the PANTHER classification system.

For each one of the 464 identified proteins a set of details can also be found, including for example its molecular function, the complete protein name or its subcellular localization. Moreover, in each component we detail the original study describing its recruitment to SGs, as well as the type of stimulus used to induce SG assembly and the type of cell employed in that study. Due to the importance of RBPs to the nucleation and formation of SGs we also detail if the identified component is an RBP or not. Interestingly and as expected, from the 464 proteins identified 252 (54%) are classified as RBPs according to Castello *et al.*’s (25) study and to the Uniprot database. In line with this data, we performed a molecular function analysis of the 464 SG components (using the Protein Analysis Through Evolutionary Relationships available at http://www.pantherdb.org), which revealed that majority of the identified proteins have a binding or catalytic activity (Figure 2).

### Online interface

**Figure 3:**
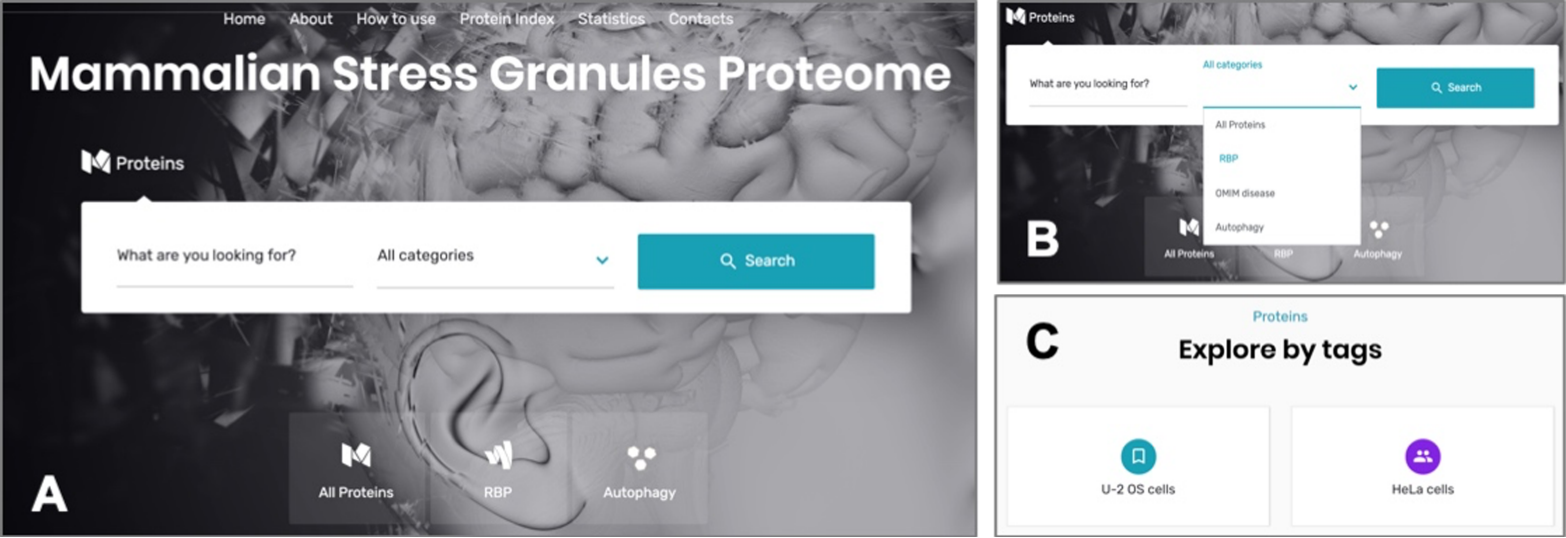
**(A)** The online interface of the database, detailing the landing page with the search form and the different pages. **(B)** The different options to narrow the search. **(C)** The possibility of accessing the listed components through different tags.

The online interface of the MSGP database homepage has a ‘Search’ form and three main tabs, ‘All Proteins’, ‘RBP’ and ‘Autophagy’ (Figure 3A). The ‘Search’ form allows a free text exploration of possible components of the database; the tab ‘All proteins’ lists all the proteins listed in the database; the tab ‘RBP’ lists the proteins classified in the database as RBPs; the tab ‘Autophagy’ groups all the proteins involved in this pathway (26) that are recruited to SGs. In the search form, four categories can be selected: (i) All Proteins, (ii) RBP, (iii) OMIM disease and (iv) Autophagy (Figure 3B). The first option allows a search in the entire database, whereas the three other options narrow the search to the SG components that are RBPs, linked to a human disease or to autophagy, respectively. The database landing page also has additional filters and tags that can be used to refine the listed proteins in the database (Figure 3C). For example, the ‘U-2 OS cells’ tab at the end of the page groups the SG components that were identified in these cells. As already mentioned, all the proteins in the database are listed in alphabetical order in the page ‘Protein Index’ to facilitate the search and to collect in the same page all the components of the SGs in the database (Figure 3A).

### Searching and search results

The components listed in the database can be searched using the most common alias for the protein, which could be found in databases such as Uniprot or GeneCards. For example, for the core SG component GTPase-activating protein (SH3 domain) binding protein 1 the search should be performed using the alias ‘G3BP1’. If no selection is made, the search is performed in the ‘All Proteins’ category (Figure 3B). Each component must be searched alone, as the use of ‘AND’, ‘OR’ and ‘NOT’ is not recognized by the database. If several SG components are listed with similar names, as in case as they belong for example to the same family, the search will list all those proteins. For example, the search for ‘PABP’ in the database will result in three hits: PABP1, PABP3 and PABP4.

### Specific component pages

**Figure 4:**
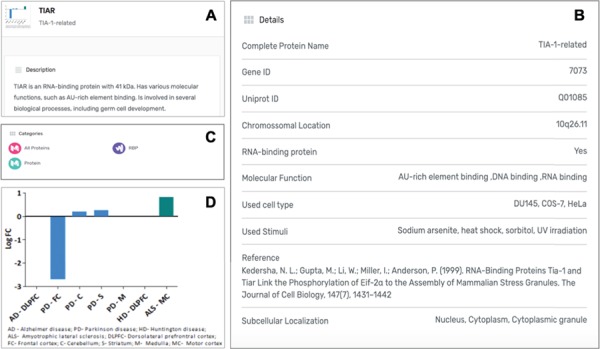
**(A)** Information details described in the specific page for the SG component TIAR, including a brief description of its molecular function. **(B)** Several details for the component are listed in the database, including the study describing the recruitment of this protein to SGs. **(C)** Each SG components is also included in specific categories, which are also displayed in its specific page. **(D)** For each SG component its expression levels in the context of neurodegenerative diseases are also described in the form of a graph, based on a differential expression analysis.

Each protein listed as a SG component in the MSGP database has a specific page where the different information details are listed. Independently from the form used to find a specific protein in the database (search, tabs, filters, index or general sections), the individual page for each protein is the same (Figure 4A). The complete name of the protein, the gene and Uniprot IDs, the chromosomal location, RBP classification (yes or no), the molecular function, OMIM details and subcellular localization are detailed for each component (Figure 4B). Each page also describes the category/categories where that specific component was classified (Figure 4C). Importantly, the original study describing the recruitment of that protein to SGs is referred, as well as the type of stimulus used to induce SGs assembly and the type of cell used in the study. In each SG component page it is also possible to find the gene expression values for that component in the context of different neurodegenerative diseases (Figure 4D).

### Expression data

**Figure 5:**
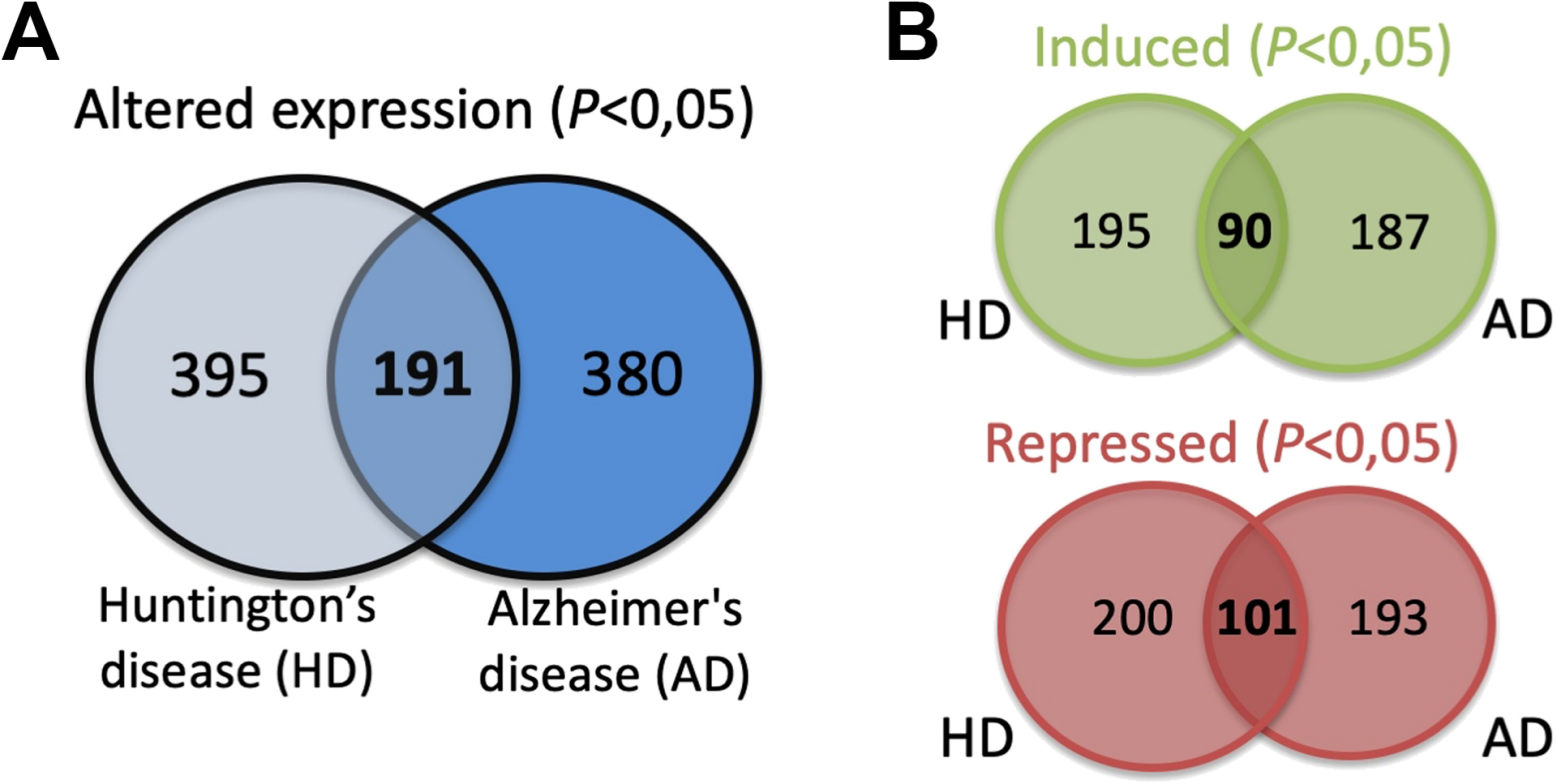
**(A)** SG components whose expression is significantly altered in the brain of AD and HD patients and commonly in both diseases. **(B)** Details on the number of SGs protein components whose expression is significantly induced or repressed in each disease and in both diseases.

The expression data for each of the SG components listed was extracted from different published studies and available in open access databases. The analysis compared the expression levels in patients with different neurodegenerative diseases and healthy controls. The differential expression level for each SG component in the different neurodegenerative diseases was plotted into a graphic and is also a part of the component-specific page (Figure 4D). As mentioned, several studies implicate SGs in different neurodegenerative diseases. Moreover, most of SG components are RBPs, which in the context of neurons are involved in different processes such as alternative splicing, transport, localization and stability and translation of RNAs (29). Thus, alterations in their expression may underlie or have impact on the neurodegenerative pathogenesis. In fact, the analysis of the differential expression of the 464 SG components detected that 380 components have the expression altered in the brain of AD patients (Figure 5A). Similarly, in the brain of HD patients, 395 have their expression significantly altered. From these, 191 SG components have their expression commonly altered in AD and HD, with 90 having the expression increased and 101 repressed (Figure 5B).

### Users interaction and future updates

At the moment the database does not have a page for users to submit new SG components; however, researchers could send that information to the contacts in the database and shortly it will be updated. Nevertheless, there will be a continuous updating for new SG components from the published studies indexed in PubMed. The entire data set of the database is also available for any researcher upon request by email to cdnobrega@ualg.pt. The future plans for updating the database include adding information on the gene expression levels of SG components for different types of cancer. Importantly, we will continue to complete each protein details with relevant information, especially SG-related, as for example the number of studies where that particular component was described. We also consider to include other SG components besides proteins, such as miRNAs or mRNAs.

## Conclusions

The MSGP database is the first tool cataloging all the SGs’ protein components described so far. Moreover, it also collects several details about each component, thus providing an important tool for researchers in this area. The growing interest in the SG field and their implication in different human diseases make this database actual and opportune. Furthermore, the MSGP database has the possibility of being continuously updated as more components are described in SGs, and also of being expanded by adding more information and details, for example detailing the expression levels of these components in the context of different types of cancer. The database will constitute an important asset for SG research and will be continuously improved, based on our already defined plans and on the feedback from users.

## Notes

**Database URL**: https://msgp.pt/
